# Editorial: Central nervous system acting drugs-molecular mechanisms of neuroprotection and neurodegeneration

**DOI:** 10.3389/fnins.2022.1060515

**Published:** 2022-10-18

**Authors:** Nesrine S. El Sayed, Riham Salah El Dine, Barbara Budzyńska, Anna Boguszewska-Czubara, Jolanta Kotlińska

**Affiliations:** ^1^Department of Pharmacology and Toxicology, Faculty of Pharmacy, Cairo University, Cairo, Egypt; ^2^Department of Pharmacognosy, Faculty of Pharmacy, Cairo University, Cairo, Egypt; ^3^Independent Laboratory of Behavioral Studies, Medical University of Lublin, Lublin, Poland; ^4^Department of Medical Chemistry, Medical University of Lublin, Lublin, Poland; ^5^Department of Pharmacology and Pharmacodynamics, Medical University of Lublin, Lublin, Poland

**Keywords:** neurodegeneration, oxidative stress, neuroinflammation, neuroprotection, molecular mechanisms

In the current world, humans are continuously exposed to different compounds that can exert deleterious effects within their bodies, notably, by changing the activity of their brains through neurodegeneration and neuroprotection. Neurodegenerative diseases are an increasingly important issue in our society. There are, however, still many obstacles in the way of finding methods for their cure. Various types of biological mechanisms have been associated with neurodegeneration, including oxidative stress, neuroinflammation, insulin signaling, mitochondrial function, iron homeostasis, and excitotoxicity. Activation of these mechanisms leads to long-term programmed cell death, whereas their blockade may improve central nervous system function, i.e., neuroprotection.

As there is currently no effective treatment for neurodegeneration resulting from both abuse of psychoactive drugs and age-dependent brain diseases, a significant part of current research effort has shifted toward finding preventive, as well as remediation treatments for neurodegeneration.

The need for effective and well-tolerated remedies for neurodegeneration has led scientists to analyze thoroughly the mechanisms of neurodegeneration and the possibility of using new drugs for the treatment of neurodegenerative diseases.

In this Research Topic, we aimed to discuss the molecular mechanisms of neurodegeneration and neuroprotection. The intention of creating this missive is to provide a thorough look into neurotoxicity induced by drug abuse, as well as the pharmacological and chemical properties, safety aspects, and interactions of new medicines for neurodegeneration treatment. We welcomed original research, and systematic reviews covering, but not limited to, the following themes:

- Overview of neuroprotective drugs that are currently in research and development to give readers ideas about the complexity of drug discovery in this field;
- Neurodegenerative mechanisms of synthetic substances, including illicit drugs;
- Molecular mechanisms of action for neuroprotective agents.

A brief discussion of the submitted papers follows.

On seizure score, as well as numerous biochemical markers, temozolomide (TMZ) has demonstrated a promising anti-seizure capability. El Shorbagy et al. have revealed that the curative effects of TMZ might be related to its capacity to reduce glutamate buildup and mitochondrial oxidative damage, in addition to a favorable regulation of p-ERK1/2/p-AMPK signaling. Moreover, TMZ administration was successful in minimizing astrocyte activation and ATP-dependent energy disturbances. All of this was demonstrated by a decrease in neuronal apoptosis and the preservation of cellular integrity.

In a ground-breaking hypothesis, Wu et al. suggest that demyelination occurs during the pathogenic Perioperative Neurocognitive Disorder (PND) phase. By inhibiting the overactivation of the WNT/-catenin signaling pathway through anti-neuroinflammation to promote oligodendrocytes (OLs) development and remyelination, the study was capable of pinpointing the ameliorative impact of clemastine on PND. At the same time, mature hippocampus neuron survival and synaptic plasticity both significantly increased. The findings may have practical ramifications and offer fresh insights and suggestions for the therapeutic management of PND.

The behavioral, histological, cellular, as well as neurochemical outcomes of the current investigation by Mustafa et al. provide evidence for the potential role of inhibition of brain GTP cyclohydrolase I in Huntington's Disease, by confirming the activation of the MasR/PI3K/Akt/CREB/BDNF/TrKB pathway and suppression of iNOS in the neuroprotective effect of 2,4-diamino-6-hydroxypyrimidine against neurotoxicity and mitochondrial dysfunction induced by 3-nitropropinic acid.

Lee et al. demonstrated the effect of HEXA-018, a novel compound containing a catechol derivative structure, as an inducer of autophagy. HEXA-018 suppressed neuronal toxicity in cell and *Drosophila* models observed as neuronal damage and behavioral impairment. These findings raise the prospect that HEXA-018-mediated amyotrophic lateral sclerosis activation might represent a cutting-edge therapeutic approach for treating neurodegenerative disorders with TDP-43 proteinopathy.

Ge et al. found that asaronol, a major compound isolated from the Chinese medicinal herb *Acorus gramineus*, promoted oligodendrocyte precursor cell (OPC) differentiation and myelination in the corpus callosum of preterm white matter injury (PWMI) rats induced by hypoxia-ischemia. They revealed that glutamate was significantly decreased, and the levels of PPARγ and glutamate transporter 1 (GLT-1) were increased by asaronol treatment. Thus they suggested that PPAR-GLT-1 mechanism mediates the effect of asaronol on OPC differentiation and myelination. Moreover, Ge et al. concluded that as asaronol could control PPAR-GLT-1 signaling it can be applied to treat myelin-related diseases.

Fluoxetine, an anti-depressive drug, has regulatory effects on autophagy and phagocytosis, which are necessary functions for microglia. Park et al., found that fluoxetine treatment causes autophagic activation, as seen by increased LC3-II and LC3 punctate distribution and autophagosome accumulation. Furthermore, the fluoxetine-mediated increase in phagocytosis was blocked by the autophagy inhibitor Baf in microglia. These findings imply that fluoxetine acts as an anti-inflammatory and neuroprotective agent in the brain *via* microglia and that altering the autophagy-lysosomal pathway may be a viable treatment for the removal of amyloid plaques in Alzheimer's disease.

A research article by Zheng et al. reported that daidzein has therapeutic potential for brain damage brought on by ischemia/reperfusion. The findings show that daidzein stimulates neural regeneration after ischemic stroke by upregulating Akt/CREB and boosting BDNF expression, albeit the potential mechanism and viability of long-term usage need to be verified. Daidzein is thought to have the potential to be a novel drug utilized in the treatment of localized cerebral ischemia.

Zhang et al.'s review provided an overview on lipoxins neuroprotective effect. When it comes to neurological conditions including ischemic or hemorrhagic stroke, newborn hypoxia-ischemia encephalopathy, brain, spinal cord injuries, Alzheimer's disease, multiple sclerosis, chronic cerebral hypoperfusion, as well as neuropathic pain; lipoxins can have a variety of protective and beneficial effects. They also have considerable therapeutic promise for neuroinflammatory and neurodegenerative ailments.

The active ingredients of bee venom have the ability to pass through the blood brain barrier and can therefore be used in the treatment of diseases of the central nervous system. Unfractionated bee venom has a dose- and time-dependent impact on glial cancer cell ability to survive. Additionally, Malek et al. revealed that the secretion of metalloproteinases MMP-2 and MMP-9 is inhibited, which could have an effect on how quickly a tumor spreads.

The development of innovative therapies for methamphetamine addiction, based on enhancing the functioning of dopamine D2-type receptors, was described by Okita et al. They revealed that upregulation of D2-type receptor and/or attenuation of neuroinflammation may provide a therapeutic effect for this disorder. *In vitro* studies have shown that blockage of adenosine 2A (A2A) receptors may prevent D2-receptor downregulation and neuroinflammation-related brain damage.

The study of Aldabbagh et al. supports the idea that Alzheimer's disease pathogenesis in the hippocampus is linked to an increased GABA content in reactive astrocytes. Astrocyte-specific GABA transporter 3/4 (GAT3/4) and expression of GAD67, an enzyme that catalyzes GABA production is altered in *APP* knock-in mouse model of Alzheimer's disease, which, may lead to an intensified tonic inhibition in the hippocampus. The mechanisms by which GAT3/4 contributes to modulating tonic inhibition are complex. Since bath-application of SNAP-5114, GAT3/4 inhibitor worsened AD-related synaptic hyperactivity. Thus, further studies are required.

Compelling evidence presented by Frank et al. confirmed that the pyruvate-induced blood glutamate scavenging mechanism results in the induction of antidepressant effects. These mechanisms prevent or treat anhedonia and hyperlocomotion, which are brought on by glutamate dysregulation following traumatic brain injury in rats. The work by Frank et al. confirmed that blood glutamate scavengers should be considered a possible therapeutic option for post-traumatic brain injury depression.

Through the research article of Liu et al., dexmedetomidine delivered in a loading dose can significantly inhibit intraoperative neuromonitoring parameters in thoracic spinal decompression surgery. Special attention should be paid to the timing of a bolus dose of dexmedetomidine under intraoperative neuromonitoring. However, dexmedetomidine delivered at a constant speed does not exert inhibitory effects.

Leite et al., in their review, deduced that cardiotonic steroids like ouabain have the ability to mobilize Ca^2+^. In their work, an increase in Ca^2+^ was described in all models that were investigated, including synaptosomes, brain slices, and cell cultures. Other effects were also observed, as Ca^2+^ may be involved in significant cellular impacts, mostly *via* activating signaling pathways. In addition to the well-known cytotoxic effects of ouabain, which occur from activation of the Na^+^/Ca^2+^ exchanger reverse mode and elevated Ca^2+^, cholinergic, noradrenergic, and glutamatergic neurotransmission might all be increased by oubabin-induced Ca^2+^ signaling. Authors suggest that ouabain therapy can dramatically enhance biological second messengers, among other biological processes. The actions and signals that cardiotonic steroids (ouabain) mediate in the nervous system (which has been demonstrated to be concentration-dependent) are more understood after the lecture of this review. The paper concludes that the development of novel, less toxic drugs with neuroprotective properties may benefit from structural alterations of the cardiotonic steroids.

Wang et al. revealed that the neuroprotective effects of epigallocatechin-3-gallate (EGCG), a bioactive ingredient in green tea, against cerebral ischemia/reperfusion injury (CIRI) are related to its ability to inhibit autophagy *via* phosphorylating AKT/AMPK/mTOR. Moreover, the research offers a fresh understanding of the possible processes underlying EGCG's influence on autophagy and cerebral ischemia damage, and it may be used to develop more effective stroke treatment plans.

Liu et al. demonstrate conclusively that akebia saponin D (ASD) enhances neural stem/precursor cells proliferation, survival, and neuronal development *via* activating the PI3K-Akt pathway and shielding them from the microglia-mediated inflammatory microenvironment. The findings suggest the need for additional research into ASD's potential as a therapeutic option for illnesses such as Alzheimer's disease, major depressive disorder, and other disorders with reduced neurogenesis.

Zhou et al. confirmed through the published review that antipsychotic medications, especially olanzapine, cause hypothalamic endoplasmic reticulum (ER) stress that is linked to inflammation and weight gain. According to the paper, antipsychotics appear to cause hypothalamic (ER) stress through the following mechanisms: (1) blocking proopiomelanocortin processing, attenuating leptin signaling, and upregulating neuropeptide Y and agouti-related protein expression, which results in hyperphagia; (2) reducing white adipose tissue browning and brown adipose tissue thermogenesis, which minimize energy expenditure; and (3) activating the MyD88-independent and MyD88-dependent pathways in astrocytes, which increases the release of pro-inflammatory cytokines. According to the review published by Zhou et al., antipsychotic-induced ER stress and the ensuing inflammation may be connected to the antagonistic interactions between histamine H1 receptor and dopamine D2 receptor and antipsychotics. When taken as a whole, hypothalamic ER stress may be a useful target for reducing the metabolic adverse effects of schizophrenia and antipsychotics, such as obesity and weight gain.

In the work of Deng et al., in rat and mouse models of traumatic spinal cord injury, high mobility group box-1 (HMGB1)-targeted treatment was found to enhance locomotor function recovery, lower inflammation attenuate edema, and lower apoptosis. The best therapy suggested by Deng et al. may be an intrathecal injection of anti-HMGB1 Ab 0–3 h after spinal cord injury. However, the low methodological caliber of the included studies severely restricted the applicability of this meta-analysis.

The presented research shows a great interest of scientists in an interdisciplinary approach of broadly understood neurodegeneration, aimed at both the discovery of molecular, cellular and biochemical mechanisms of emerging changes as well as the development of innovative therapies based on new synthetic substances and naturally obtained from the environment (from plants, bacteria, animals, etc.).

## Author contributions

All authors listed have made a substantial, direct, and intellectual contribution to the work and approved it for publication.

## Conflict of interest

The authors declare that the research was conducted in the absence of any commercial or financial relationships that could be construed as a potential conflict of interest.

## Publisher's note

All claims expressed in this article are solely those of the authors and do not necessarily represent those of their affiliated organizations, or those of the publisher, the editors and the reviewers. Any product that may be evaluated in this article, or claim that may be made by its manufacturer, is not guaranteed or endorsed by the publisher.

